# Thoracic aortic aneurysm. An experimental model in pigs

**DOI:** 10.1590/ACB360602

**Published:** 2021-06-25

**Authors:** Rodrigo Argenta, Sílvio César Perini, Adamastor Humberto Pereira

**Affiliations:** 1 MD. Postgraduate Program in Surgical Sciences - Medical Faculty - Universidade Federal do Rio Grande do Sul - Porto Alegre (RS), Brazil.; 2 MD. Postgraduate Program in Surgical Sciences - Medical Faculty - Universidade Federal do Rio Grande do Sul - Porto Alegre (RS), Brazil.; 3 Associate Professor. Medicine Faculty, Universidade Federal do Rio Grande do Sul. Head. Vascular Surgery Department - Hospital de Clínicas de Porto Alegre - Porto Alegre (RS), Brazil.

**Keywords:** Aortic Aneurysm, Thoracic, Models, Animal

## Abstract

**Purpose:**

To describe an unpublished experimental model of descending thoracic aortic aneurysm in pigs.

**Methods:**

Ten Landrace female pigs aged 10 to 12 weeks old and with initial weights from 17 to 25 kg were anesthetized and their descending thoracic aortas exposed by fifth intercostal space left thoracotomy. The thoracic aorta was isolated. A 2-cm wide × 2-cm long patch of ready-made bovine pericardium was sewn onto the left anterolateral side of the aorta. After three weeks’ follow-up, a control aortography was taken, and the animals were euthanized. The segment of thoracic aorta containing the aneurysm and the adherent tissues were explanted en bloc. The specimens were stained for histological examination.

**Results:**

One hundred percent of the animals survived the procedure, and after sacrifice a patent aneurysm was observed in all of them. There were no defects on the suture lines. Weight gain during follow-up was normal. All specimens exhibited intense adventitial reaction with myofibroblasts. There were no complications related to the thoracotomy.

**Conclusions:**

The descending thoracic aortic aneurysms induced experimentally appear to be stable, were of easy execution, with null mortality and no influence on the animals’ normal development. Furthermore, they have similar characteristics to those observed in human degenerative aneurysms.

## Introduction

Experimental animal models have been used for decades in studies of the natural history of arterial aneurysms and in the evaluation of the results of treating them^1^. These models differ in terms of some of their characteristics, such as the size of experimental animal, the way the aneurysm is produced and its location, in addition to other details. Nevertheless, the objective of these models is always the same: to mimic the way that aneurysmatic pathologies appear in humans.

Some models approach this ideal when specimens are analyzed histologically^2^,^3^, others when we observe the changes in diameter and the tendency towards rupture^4^,^5^, and yet others in terms of the dimensions of vessels and aneurysms^6^,^7^. However, there is no model with all of the characteristics observed in human arterial aneurysm^8^,^9^.

There are several experimental models of aortic abdominal aneurysms^10^, but models for the study of thoracic aortic aneurysms (TAA) are rare^11^,^12^.

A murine model has been developed based on induction of TAA using elastase^12^. Although this is an excellent model for studying the histology of aneurysms, the small size of the animal limits its applicability. Another model was developed using dogs, in which a polyester patch was sewn onto the anterior side of the thoracic aorta^11^. That study provided a comprehensive investigation of the healing processes associated with the Cragg EndoPro System 1. However, the structure and morphology of the human arterial wall are different from the ones of dogs, and pathological analysis of the aneurysm was not described.

The superior results obtained with endovascular treatment of pathologies of the descending thoracic aorta, owing to the development of new stent-graft devices and endovascular procedures, mean that in the majority of cases this is the technique of choice for treating TAA in section of the aorta^13^.

This new technology, however, demands particular skills, both from surgeons in training and from those already qualified for some time. Training these people using in-vitro or robotic models has not proven to be as realistic as when animal models are employed^14^,^15^.

Considering all of that, we proposed the development of an experimental model of descending TAA in pigs which exhibits anatomic and histopathological characteristics similar to the human aneurysm, for use in training surgeons and in the development of new endovascular devices.

## Methods

This cross-sectional experimental study was conducted at the Animal Research Center at the Hospital de Clínicas de Porto Alegre, Universidade Federal do Rio Grande do Sul (Porto Alegre, RS, Brazil). All animals were treated and cared for in accordance with regulations of the Guide for the Care and Use of Laboratory Animals, and all protocols were approved by the Research Ethics Committee at the Hospital de Clínicas de Porto Alegre.

We created thoracic aortic aneurysm in 10 female 10 to 12-week-old Landrace pigs and initial weights from 17 to 25 kg, sourced from a local supplier (Agrogen, Montenegro, RS, Brazil). The animals were fed with standard diet for their age and underwent a 12-hour fast before the procedure.

One animal was maintained on the regular diet for three weeks, without intervention, to enable comparison between its weight gain and development and the ones of the animals submitted to the induced TAA. Another animal was submitted to TAA induction and euthanized 24 hours later (pilot study).

The 10 pigs with TAA were euthanized three weeks after the initial procedure. The thoracic aorta containing the TAA was harvested and fixed in formalin. After setting in paraffin, they were prepared for optical microscopy.

### Experimental procedure

After 12 hours’ fasting, animals were weighed and given intramuscular sedation with midazolam (1 mg/kg). At this point, after disinfection, venous access was obtained by puncture of the marginal vein of the ear with a 16G teflon catheter (Abocath). Hydroelectrolytic replacement and intravenous medications were delivered via this route.

**Figure 1:**
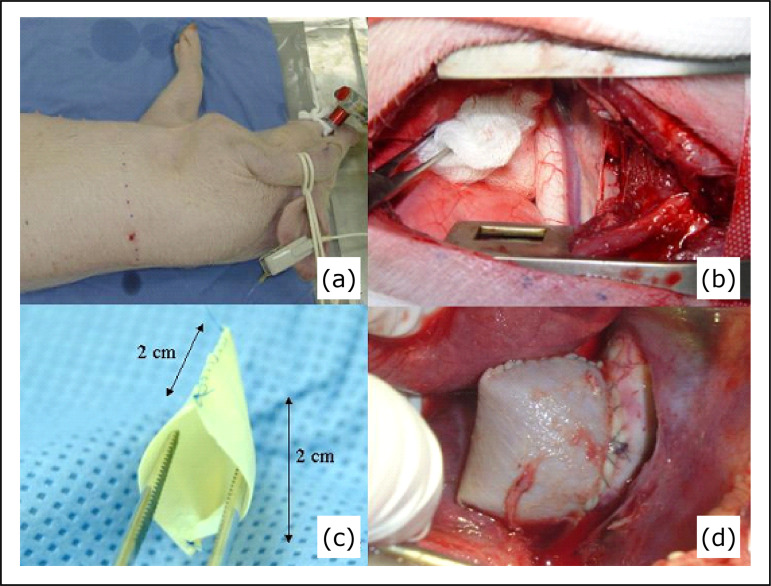
**(a)** Animal positioning. **(b)** Thoracic aorta exposure. **(c)** Bovine pericardium pouch. **(d)** Aneurysm sac after releasing clamps.

The animals were placed in the supine position, and orotracheal intubation was performed. Anesthesia was maintained with 2-mg intravenous ketamine and 2%-isoflurane in combination with oxygen FiO2 100%. At this point, 2 g of cephazolin was administered intravenously for prophylaxis. During the procedure, hydroelectrolytic balance was maintained with 20 mL/kg/h of 0.9%-NaCl solution intravenously. Mechanical ventilation was provided by a closed-loop re-inhalation system (Husky, Calgimed). The pigs were monitored using pulse oximetry and electrocardiography (Fig. 1).

For best visualization, a saccular structure was made, with bovine pericardium (11 × 6 × 0,52 mm) (Braile Biomedica, São José do Rio Preto, SP, Brazil). The final size was 2 × 2 cm, suturing the lateral edges with polypropylene 6 stitches.

As we planned a histological examination of the aneurysm, the explant of the piece was made.

After general anesthesia, animals were placed on their right lateral sides, and the left hemithorax was prepared in a sterile manner. The fifth intercostal space was infiltrated with bupivacaine at 0.5% as a supplementary anesthetic (Fig. 1).

A thoracotomy was then performed opening onto the pleural cavity. Retraction of the left lung permitted the descending thoracic aorta to be located and isolated and the intercostals arteries to be identified and controlled with vessel loops.

Following an intravenous injection of heparin (100 U/kg), the thoracic aorta was clamped. A 2-cm aortotomy was created, and the pericardium pouch was sewn on with a continuous polypropylene monofilament suture. After anastomosis, the clamps were removed, and the suture line was examined.

After this step, the thoracotomy was closed. During the thoracoraphy, the lung was maintained hyperinflated, and a rigid silicon drain was located in the pleural cavity. This drain was removed during final closure.

After recovery from the anesthetic, the animals were kept under observation by the veterinary team at the Research Center, analgesia being given as needed. After 24 hours, the animals were transferred to a farm and observed for 20 days, with regular diet.

### Artheriographic control and euthanasia

After three weeks, the animals were weighed, and the anesthetic protocol was applied. The left hemithorax and left groin were prepared.

**Figure 2:**
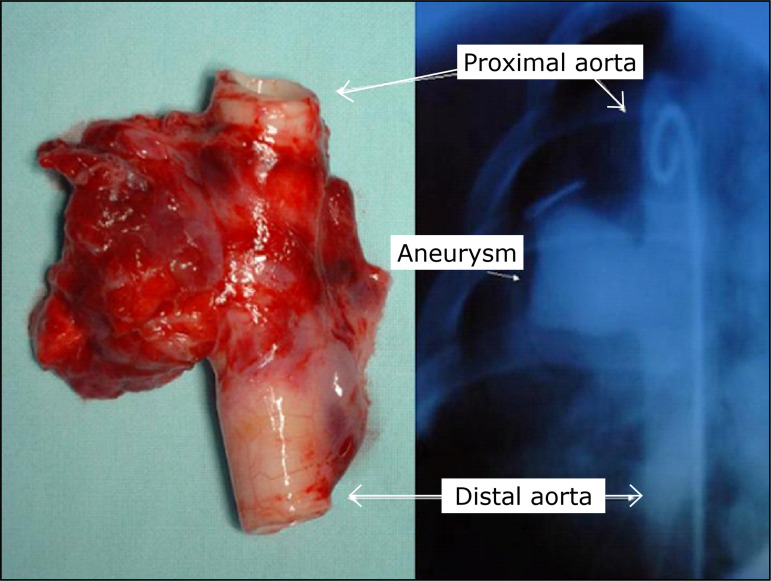
Descending thoracic aorta with aneurysm and control aortography.

After infiltration of bupivacaine at 0.5%, the left common femoral artery was dissected and, under direct view, a valved 5F shaft was fitted. Through the shaft, a diagnostic pig-tail 5F catheter was placed in the thoracic descendent aorta, over a 0.035” metal wire. Conventional aortography was then carried out after injection of 20 mL of iodinated contrast (Hypaque), in order to confirm the patency of the aneurysm (Fig. 2).

### Anatomopathological analysis

After the thoracotomy and control of the thoracic aorta, as described, a lethal intravenous dose of KCl was administrated, and the segment of the descendent thoracic aorta containing the aneurysm was explanted en bloc. The excised aorta and aneurysm were cleaned in saline solution and fixed in 10% neutral buffered formalin.

The specimens were photographed, and selected areas were embedded in paraffin. The 5-μm-thick slices were representative of the aneurysm circumference and suture line. They were stained with hematoxylin-eosin, Masson and anti-human actin immunohistochemical (clone HHF35) stains.

Qualitative observation was carried out and the following features recorded, if observed: mural thrombus, endothelization, transmural and periadventitial inflammatory reaction, and calcification. In the macroscopic evaluation, the integrity of the suture line, surrounding tissue adhesions and patency of the intercostals arteries were all assessed.

### Definitions and statistical analysis

Aneurysm was defined as a focal dilation of at least 50% of the proximal diameter of the adjacent thoracic aorta. It was measured using aortography and confirmed by the measuring the specimen during the macroscopic examination.

To illustrate the animals’ development during the study period, their mean initial weight was compared with their mean final weight, at the time of euthanasia. These values were compared with descriptions in specific articles^17^,^18^ and with the control animal.

The sample size was estimated based on an average of similar studies carried out previously.

The results obtained were expressed as mean ± standard deviation. Student’s *t* test for paired samples was used to compare weight, considering significant p < 0.05.

## Results

### Experimental model

All 10 animals submitted to the aneurysm induction procedure survived. No major complications such as paraplegia or paraparesis were observed during the follow-up. Other complications, such as infected wounds, seromas or hematomas, were not observed either.

The mean operating time was 46.1 ± 7.6 minutes, and the mean clamping time was 11.9 ± 2.3 minutes.

All animals exhibited tachycardia while the thoracic aorta was clamped, but spontaneously recovery occurred after the clamps were released.

After extubation, five animals (50%) suffered airway spasms, also recovering spontaneously within minutes, without compromising oxygen saturation.

All animals presented patent aneurysms at the time of control aortography.

At the time of euthanasia, the segment of the thoracic aorta containing the aneurysm was firmly attached to the adjacent lung tissue in all the cases, and none of the animals exhibited pleural effusion at this point.

**Figure 3:**
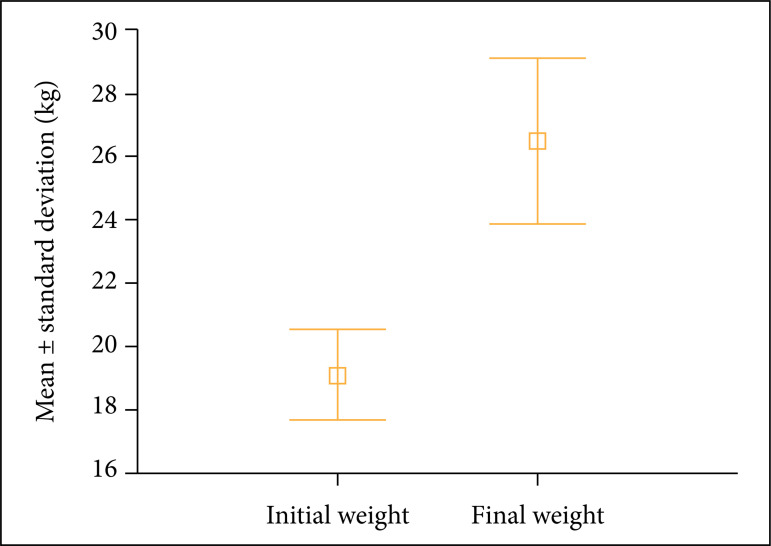
Weight gain during follow-up. Difference between groups, Student’s t test for paired samples (p < 0.05).

All animals gained weight during the 21 days of follow-up. The initial mean weight was 19.1 ± 2.2 kg. At euthanasia, mean weight was 26.5 ± 4.1 kg (p < 0.05) (Fig. 3). The experimental group exhibited a mean daily weight gain of 352 g, varying from 80 to 523 g. The control animal’s weight increased from 19 to 28 kg in the same period of time, with a daily gain of 428 g.

### Macroscopy and histology

**Figure 4:**
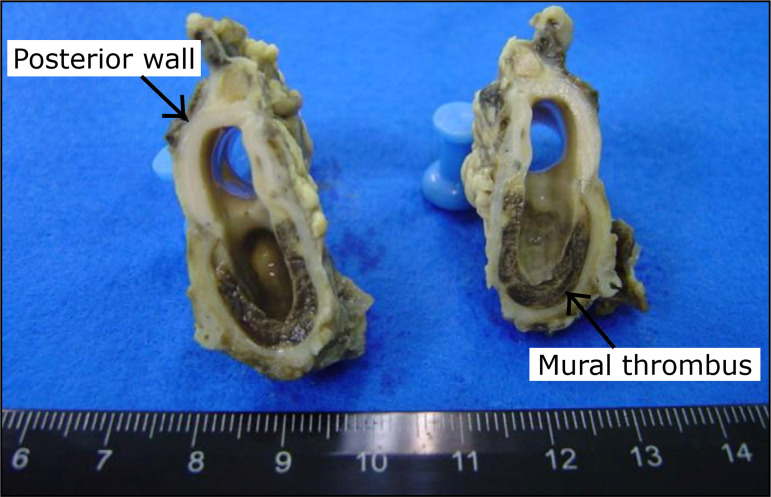
Aneurysm after conservation in buffered formalin.

Each specimen consisted of a segment of the thoracic aorta containing the aneurysm, the adjacent connective tissue and adhering lung parenchyma (Fig. 4).

The mean diameter of the descending thoracic aorta, proximal to the aneurysm, was 1.1 ± 0.2 cm, and the mean of maximum diameter observed at the aneurysms was3.1 ± 0.2 cm (p < 0.05).

All specimens contained patent intercostal arteries adjacent to the aneurysm.

**Figure 5:**
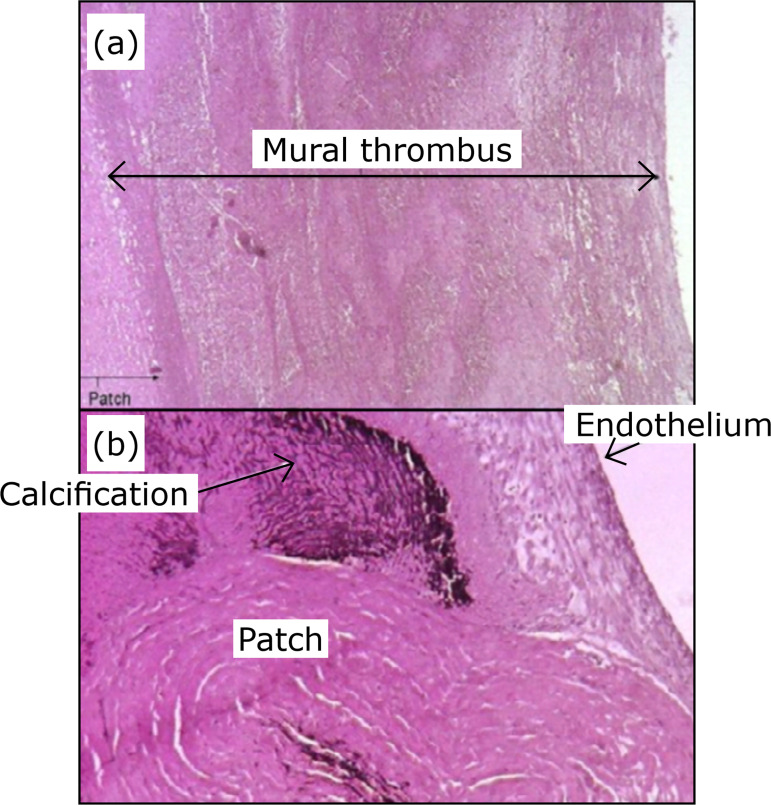
Hematoxilin-eosin stain. (**a**) Mural thrombus (100x). **(b**) Suture line calcifications (250x).

Endothelization occurred in all specimens. In five cases, there was complete covering of the aneurysmal sac by endothelium; in the other five, only partial covering. Mural thrombi were observed in eight cases (80%). In one case, with total endothelization, there was no mural thrombus. In four specimens (40%), intrathrombus calcification occurred, and in all cases calcification was seen at the suture line (Fig. 5).

There were granulomatous reactions in the perianeurysmatic region and also at the native aorta.

**Figure 6:**
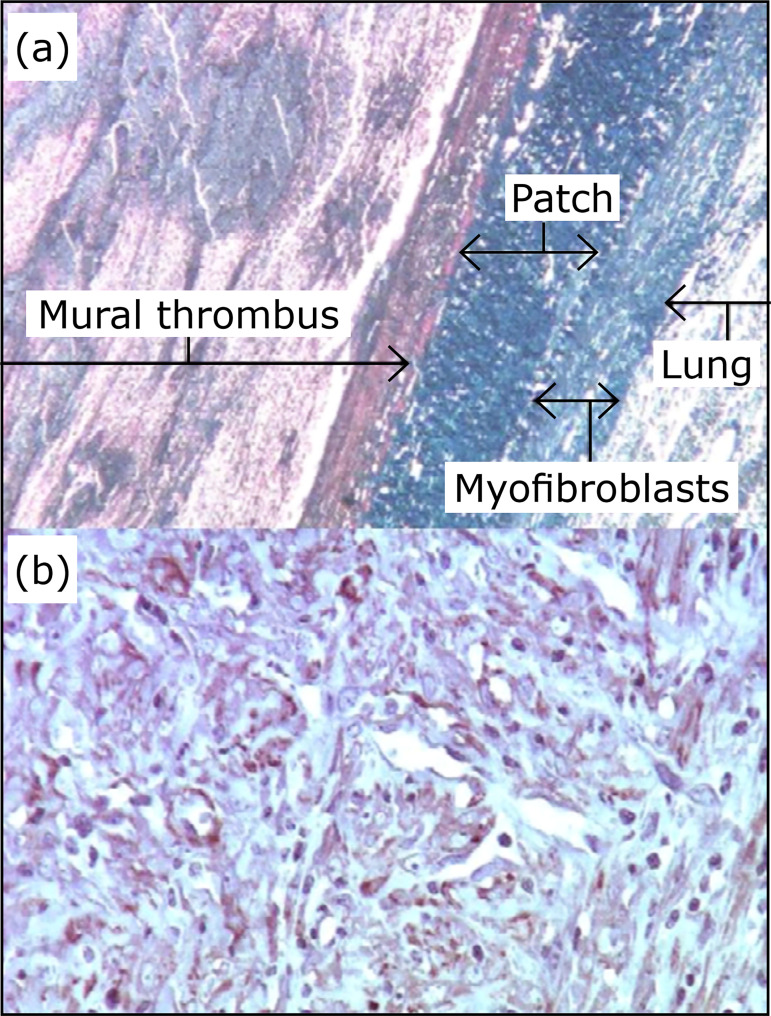
**(a**) Healing process in the aneurysm wall (Masson, 250x). **(b**) Periadventitial myofibroblasts, actin positive (clone HHF35, 400x).

Actin-positive smooth cell infiltration occurred at the periadventitial region in all cases. The thickness of this layer was variable, but significant (Fig. 6).

## Discussion

Thoracic aortic aneurysm and aortic dissection are the principal pathologies related to this artery. Mortality as high as 34% was seen in the first 30 days after intervention^19^-^21^. A recent publication reported the incidence of these pathologies as being higher than had previously been described^20^, reaching 16.3 cases per 100,000 males^22^.

The impact of endovascular intervention on descending aortic aneurysm cases is striking^20^,^21^, although comparative studies free from significant bias comparing groups of conventional and endovascular surgery have not been carried out^13^, and possibly never can be, for ethical reasons. The almost continuous development of the endoprostheses employed has contributed to achieve better results. However, the introduction of these better-quality materials must be followed by the development of better skills in those who operate with them.

From this perspective, it is necessary to make available an animal model that offers the possibility of both training professionals and testing new materials.

Experimental animal models are better than robotic or virtual models for training operators and offer the necessary conditions to simulate all steps of the procedure^15^,^24^.

Although there are several experimental models of abdominal aortic aneurysm, models of thoracic aortic aneurysm are rare^11^,^12^.

We chose pigs for this animal experimental model based on the need for ease of handling, low cost, and ethical acceptability. We know that the cardiovascular system and coagulation response of these animals are similar to humans^23^-^24^. Furthermore, there are no references in the literature to such a model using pigs.

The primary objective was to develop a surgical operation that the experimental animal could tolerate. A highly skilled operation requiring left thoracotomy could cause certain difficulties, from maintaining adequate ventilation, to pneumothorax after thoracoraphy, pleural hemorrhage and atelectasis. Another important point to be considered was the possibility that the intervention could interfere in the animal’s normal growth curve expected.

As in the canine experimental model described by Formichi et al.^11^, there were no deaths or paraplegia among our animals. The survival of all animals allows for the conclusion that this intervention is tolerable. Moreover, the animals gained the mean weight of 352 g per day. At this age, weight gain for this race is influenced by external factors and has wide limits, with 234 to 451 g to be expected per day-18. Three animals had less than the expected weight gain during the observation period. All of the others had adequate weight gain, which means that the intervention was well tolerated by the majority of the experimental subjects.

While performing the control arteriography, we observed that the animals’ femoral arteries were of small diameter and there was intense vasoconstriction making handling difficult. We therefore chose to gain access through the external iliac artery. Although there was still vasoconstriction, here the diameter was sufficient to allow the 5F to be inserted.

The control angiography did not identify the patency of the intercostals arteries, because the Animal Experimental Center did not have equipment capable of identifying them. However, patency was confirmed during removal of the aorta and macroscopic analysis.

The macroscopic appearance and the arteriographic results were very similar to observations made of an experimental model by Uflacker et al.^25^, except for two important differences. Firstly, in that model, the location of the induced aneurysm was the abdominal aorta, in which hemodynamics is significantly different from at the thoracic aorta. Second of all, the material used in the study cited was polyester, which has a different behavior from a biological material such as the bovine pericardium used in our study.

There are no references in the literature describing the microscopic characteristics of thoracic aneurysms made in experimental animals before exclusion with endoprostheses.

Complete endothelization of the aneurysm sac was observed in five specimens. In all others, endothelization was incomplete. Some abdominal and iliac aorta models that have been described underwent endothelization^27^,^28^, while others had formation of neointima^29^,^30^. The different flow characteristics of the thoracic aorta, the interval between surgical intervention and data collection and the format of the aneurysm may be responsible for this variation. Mural thrombi were observed in eight animals (80%), two of which contained calcification. It is possible that the same reasons that influenced endothelization of the aneurysms could have caused this, although no correlation was seen between endothelization and the formation of mural thrombi in this model.

The intense healing reaction with myofibroblasts in the periadventitial region is similar to the reaction described by Zollikofer et al.^30^ with relation to a dog model. This reaction is not observed in degenerative aneurysms in humans, and it has not been described in experiments with pigs. A longer observation period is needed to determine how this tissue response develops.

The follow-up period was sufficient for histopathological alterations to be observed in the area of the induced aneurysm. However, a longer follow-up period would permit further observations. However, in such case, the pigs’ rapid growth up would be a limiting factor. Genetically-modified animals with slower growth could make such a follow-up study feasible.

The model we have described here has characteristics that are observed in human aneurysms, such as preservation of intercostal arteries, presence of mural thrombi, inflammatory vessel wall reactions and calcifications. The model was stable throughout the study, and the technique was well-tolerated by the experimental animals. We believe these characteristics are sufficient to make this model a good choice as a tool for the study of new endovascular devices studies and for training in these techniques.

## Conclusion

The descending thoracic aortic aneurysms induced experimentally appear to be stable, was of easy execution, with null mortality and with no influence on the animals’ normal development.

## References

[1] Argenta R, Pereira AH (2009). Modelos animais de aneurisma de aorta. J Vas Bras.

[2] Anidjar S, Salzmann JL, Gentric D, Lagneau P, Camilleri JP, Michel JB (1990). Elastase-induced experimental aneurysms in rats. Circulation.

[3] Anidjar S, Dobrin PB, Eichorst M, Graham GP, Chejfec G (1992). Correlation of inflammatory infiltrate with the enlargement of
experimental aortic aneurysms. J Vasc Surg.

[4] Criado E, Marston WA, Woosley JT, Ligush J, Chuter TA, Baird C, Suggs CA, Mauro MA, Keagy BA (1995). An aortic aneurysm model for the evaluation of endovascular
exclusion prostheses. J Vasc Surg.

[5] Martson WA, Criado E, Baird CA, Keagy BA (1996). Reduction of aneurysm pressure and wall stress after endovascular
repair of abdominal aortic aneurysm in a canine model. Ann Vasc Surg.

[6] Mousa A, Dayal R, Bernheim J, Henderson P, Hollenbeck S, Trocciola S, Prince M, Gordon R, Badimon J, Fuster V, Marin ML, Kent C, Faries PL (2005). A canine model to study the significance and hemodynamics of type
II endoleaks. J Surg Res.

[7] Rhee JY, Trocciola SM, Dayal R, Lin S, Chaer R, Kumar N, Mousa A, Bernheim J, Christos P, Prince M, Marin ML, Gordon R, Badimon J, Fuster V, Kent CK, Faries PL (2005). Treatment of type II endoleaks with a novel polyurethane
thrombogenic foam: induction of endoleak thrombosis and elimination of
intra-aneurysmal pressure in the canine model. J Vasc Surg.

[8] Narayanaswamy M, Wright KC, Kandarpa K (2000). Animal models for atherosclerosis, restenosis, and endovascular
graft research. J Vasc Interv Radiol.

[9] Dobrin PB (1999). Animal models of aneurysms. Ann Vasc Surg.

[10] Lee LK, Faries PL (2007). Assessing the effectiveness of endografts: clinical and
experimental perspectives. J Vasc Surg.

[11] Formichi M, Marois Y, Roby P, Marinov G, Stroman P, King MW, Douville Y, Guidoin R (2000). Endovascular repair of thoracic aortic aneurysm in dogs:
evaluation of a nitinol-polyester self-expanding stent-graft. J Endovasc Ther.

[12] Ikonomidis JS, Gibson WC, Gardner J, Sweterlitsch S, Thompson RP, Mukherjee R, Spinale FG (2003). A murine model of thoracic aortic aneurysms. J Surg Res.

[13] Svensson LG, Kouchoukos NT, Miller DC, Bavaria EJ, Coselli JS, Curi MA, Eggebrecht H, Elefteriades JA, Erbel R, Gleason TG, Lytle BW, Mitchell RS, Nienaber CA, Roselli EE, Safi HJ, Shemin RJ, Sicard GA, Sundt TM, Szeto WY, Wheatley GH, 3rd, Society of Thoracic Surgeons Endovascular Surgery Task
Force (2008). Expert consensus document on the treatment of descending thoracic
aortic disease using endovascular stent-grafts. Ann Thorac Surg.

[14] Gould DA, Reekers JA, Kessel DO, Chalmers NC, Sapoval M, Patel AA, Becker GJ, Lee MJ, Stockx L, CIRSE (2006). Simulation devices in interventional radiology: caveat
emptor. Cardiovasc Intervent Radiol.

[15] Berry M, Lystig T, Beard J, Klingestierna H, Reznick R, Lönn L (2007). Porcine transfer study: Virtual reality simulator training
compared with porcine training in endovascular novices. Cardiovasc Intervent Radiol.

[16] Kerr BJ, Yen JT, Nienaber JA, Easter RA (2003). Influences of dietary protein level, amino acid supplementation
and environmental temperature on performance, body composition, organ
weights and total heat production of growing pigs. J Anim Sci.

[17] Costa ARC, Lopes PS, Torres RA, Regazzi AJ, Silva MA, Euclydes RF, Pires AV (2001). Estimação de parâmetros genéticos em características de
desempenho de suínos das raças Large White, Landrace e Duroc. Rev Bras Zootec.

[18] Manno MC, Oliveira RFM, Donzele JL, Ferreira AS, Oliveira WP, Lima KRS, Vaz RGMV (2005). Efeito da temperatura ambiente sobre o desempenho de suínos dos
15 aos 30 kg. Rev Bras Zootec.

[19] Olsson C, Thelin S, Stahle E, Ekbom A, Granath F (2006). Thoracic aortic aneurysm and dissection: increasing prevalence
and improved outcomes reported in a nationwide population-based study of
more than 14.000 cases from 1987 to 2002. Circulation.

[20] Patel HJ, Shillingford MS, Williams DM, Upchurch GR, Dasika NL, Prager RL, Deeb GM (2007). Survival benefit of endovascular descending thoracic aortic
repair for the high-risk patient. Ann Thorac Surg.

[21] Makaroun MS, Dillavou ED, Kee ST, Sicard G, Chaikof E, Bavaria J, Williams D, Cambria RP, Mitchell RS (2005). Endovascular treatment of thoracic aortic aneurysms: results of
phase II multicenter trial of the GORE TAG thoracic
endoprosthesis. J Vasc Surg.

[22] Neequaye SK, Aggarwal R, Van Herzeele I, Darzi A, Cheshire NJ (2007). Endovascular skills training and assessment. J Vasc Surg.

[23] Schwartz RS, Huber KC, Murphy JG, Edwards WD, Camrud AR, Vlietstra RE, Holmes DR (1992). Restenosis and the proportional neointimal response to coronary
artery injury: results in a porcine model. J Am Coll Cardiol.

[24] Mason RG, Read MS (1971). Some species differences in fibrinolysis and blood
coagulation. J Biomed Mater Res.

[25] Uflacker R, Brothers T (2006). Filling of the aneurysmal sac with DEAC-glucosamine in an animal
model of abdominal aortic aneurysm following stent-graft
repair. J Cardiovasc Surg (Torino).

[26] França LHG, Pereira AH, Perini SC, Argenta R, Aveline CC, Mollerke RO, Soares ME, Nóbrega F, Ferreira MP (2005). Modelo experimental de aneurisma sacular de artéria ilíaca comum
com pericárdio bovino em suínos. J Vasc Br.

[27] Soula P, Jane d’Othée, Otal P, Amin C, Khoury JE, Delisle MB, Cérène A, Joffre F, Rousseau H (2001). Macroporous polyester-covered stent in an experimental abdominal
aortic aneurysm model. J Endovasc Ther.

[28] Wisselink W, Abruzzo FM, Shin CK, Ramirez JR, Rodino W, Kirwin JD, Panetta TF (1999). Endoluminal repair of aneurysms containing ostia of essential
branch arteries: an experimental model. J Endovasc Surg.

[29] Faries PL, Sanchez LA, Marin ML, Parsons RE, Lyon RT, Oliveri S, Veith FJ (1997). An experimental model for the acute and chronic evaluation of
intra-aneurysmal pressure. J Endovasc Surg.

[30] Zollikofer CL, Redha FH, Bruhlmann WF, Uhlschmid GK, Vlodaver Z, Castaneda-Zuniga WR, Amplatz K (1987). Acute and long-term effects of massive balloon dilation on the
aortic wall and vasa vasorum. Radiology.

